# THE RELATIONSHIP BETWEEN SCHOOL ENVIRONMENT CONDITION AND DENGUE HEMORRHAGE FEVER INCIDENCE AT PUBLIC ELEMENTARY SCHOOL

**DOI:** 10.21010/Ajidv19i2.7

**Published:** 2025-04-07

**Authors:** Selamat Ginting, Hariati Hariati, Friska Ernita Sitorus, Jekson Martiar siahaan

**Affiliations:** 1 Department of Public Health, Faculty of Nursing, Institut Kesehatan Deli Husada Deli Tua, Indonesia; 2 Department of Medical-Surgical Nursing, Faculty of Nursing, Institut Kesehatan Deli Husada Deli Tua, Indonesia; 3 Department of Medical-Surgical Nursing, Faculty of Nursing, Institut Kesehatan Deli Husada Deli Tua, Indonesia; 4 Department of Physiology, Faculty of Medicine, Institut Kesehatan Deli Husada Deli Tua, Indonesia

**Keywords:** Dengue incidence, school environment, elementary school, public health

## Abstract

**Background:**

Dengue cases in Indonesia remain a significant public health concern, with incidence rates increasing over the years. The school environment, including lighting, humidity, temperature, and ventilation, plays a crucial role in the spread of Dengue Hemorrhagic Fever (DHF). This study aims to analyze the relationship between school environmental conditions and DHF incidence.

**Materials and Methods:**

This was an analytical observational study with a cross-sectional design. The sample consisted of 157 individuals selected using simple random sampling. Data collection was conducted from August to October 2023 using a validated questionnaire assessing school environmental conditions and DHF incidence. Statistical analysis was performed using chi-square and logistic regression tests with a significance level of p < 0.05.

**Results:**

School environmental factors significantly associated with DHF incidence included lighting (p = 0.008, PR = 1.82), humidity (p = 0.008, PR = 1.75), temperature (p = 0.045, PR = 1.498), and screen ventilation (p = 0.000, PR = 2.26). Logistic regression analysis identified humidity (OR = 7.16; 95% CI, 2.09–38.37) and ventilation (OR = 18.12; 95% CI, 3.36–56.70) as the most influential factors.

**Conclusion:**

The incidence of DHF is closely related to school environmental conditions. Preventive measures focusing on environmental cleanliness and improved ventilation could significantly reduce the risk of dengue transmission among school children.

## Introduction

Dengue Hemorrhagic Fever (DHF) remains a major public health challenge in Indonesia, with incidence rates showing a persistent upward trend. Despite ongoing prevention efforts, public awareness regarding early detection and management of DHF remains inadequate, often resulting in severe complications and fatalities (Anggraini, 2018). DHF is a vector-borne disease caused by the dengue virus, primarily transmitted through Aedes mosquitoes. The first recorded case of DHF in Indonesia was reported in Surabaya in 1968, and since then, its incidence has steadily increased (Ministry of Health of the Republic of Indonesia, 2023).

Globally, approximately 2.5 billion people reside in regions endemic to dengue, with Southeast Asia among the most affected areas (WHO, 2024). The Association of Southeast Asian Nations (ASEAN) consists of 10 countries, where an estimated 1.3 billion people live in dengue-prone zones, including Indonesia (Yacoub et al., 2022). DHF is primarily transmitted through the bite of Aedes aegypti mosquitoes and is regarded as one of the fastest-spreading mosquito-borne diseases worldwide (Zhang, 2023; Atika et al., 2023; Priesley, 2018; Gregorio, 2024).

Environmental factors play a pivotal role in DHF transmission. Poor sanitation, stagnant water, and inadequate waste management create ideal conditions for Aedes mosquito breeding, thereby increasing the risk of infection. In addition to environmental determinants, behavioral factors, healthcare infrastructure, and genetic predisposition also significantly influence disease spread and public health outcomes (Ministry of Health of the Republic of Indonesia, 2021).

The World Health Organization (WHO) has reported a dramatic surge in DHF cases over the past two decades. The number of reported cases increased from 505,430 in 2000 to over 5.2 million in 2019 (WHO, 2023). Additionally, dengue-related fatalities rose from 960 in 2000 to 4,032 in 2015, predominantly affecting younger populations (Shepard et al., 2016). However, a decline in reported cases was observed in 2020 and 2021, likely due to disruptions in case reporting during the COVID-19 pandemic (WHO, 2023).

In Indonesia, DHF incidence has shown fluctuations over the years. In 2019, a total of 138,127 cases were reported, reflecting a significant increase from 65,602 cases in 2018 (Ministry of Health of the Republic of Indonesia, 2019). However, in 2020, the number of cases declined to 108,303. Specifically, in North Sumatra, 7,584 DHF cases and 37 fatalities were recorded in 2019, marking an increase from 5,786 cases and 26 fatalities in 2018. Although variations in incidence are observed, the overall trend remains concerning, as evidenced by the Case Fatality Rate (CFR), which stood at 0.5% in 2019. Given the fluctuating incidence and the ongoing threat of DHF, particularly among school-aged children, there is a pressing need to investigate environmental factors that may contribute to disease transmission.

Schools, as densely populated environments, may serve as potential breeding sites for Aedes mosquitoes, thereby facilitating the spread of DHF. Numerous studies have explored the impact of environmental conditions on DHF transmission. However, research specifically examining school environments remains limited, despite their potential role in mosquito proliferation. Previous studies have highlighted the influence of temperature, humidity, and ventilation on DHF transmission (Liliandriani et al., 2022; Monintja et al., 2021; Gregorio, 2024), yet the extent of these factors’ contributions within school settings remains underexplored. This study aims to bridge this research gap by investigating the relationship between school environmental conditions and DHF incidence. The findings will provide evidence-based recommendations for implementing targeted preventive measures in educational institutions, thereby contributing to more effective public health interventions in endemic regions.

## Materials and Method

### Research Design

This study employed a descriptive correlational design to examine the relationship between school environmental conditions and the incidence of Dengue Hemorrhagic Fever (DHF) in public elementary schools.

Participants were selected through a two-stage sampling process. In the first stage, cluster sampling was employed to divide the population into groups based on school location. In the second stage, simple random sampling was applied within each cluster to ensure a representative sample. This approach was used to minimize selection bias and improve the generalizability of the findings.

The study employed a two-stage sampling process to ensure a representative selection of participants. In the first stage, *cluster sampling* was used to categorize the total population of 234 students into groups based on their school grade levels. This method was chosen to ensure proportional representation of different grade levels in the study. Subsequently, in the second stage, *simple random sampling* was applied within each cluster to randomly select a total of 126 participants (Table 1). A lottery system was used to ensure fairness in participant selection, based on student attendance records. This combined approach minimized selection bias and enhanced the generalizability of the findings.

**Table 1:** Characteristics of respondents in the Study (n=126)

Characteristics	n (%)
**Gender**	
Male	73 (57.90)
Female	53 (42.10)
**Age**	
<10 Years	42 (33.30)
>10 Years	84 (66.70)
**Class**	
3 Class	27 (21.10)
4 Classes	51 (40.40)
5 Classes	48 (33.60)
**Lighting**	
Not eligible	77 (61.40)
Qualify	49 (33.60)
**Humidity**	
Not eligible	71 (56.10)
Qualify	55 (43.90)
**Temperature**	
Not eligible	68 (54.4)
Qualify	58 (45.6)
**Glass Vent**	
Not eligible	77 (61.20)
Qualify	49 (36.80)
**Dengue fever incidence**	
Yes	86 (68.4)
No	40 (31.6)

### Research Instruments

Several validated instruments were utilized in this study. The DHF questionnaire was adapted from Liliandri (2022) and consisted of two dichotomous (yes/no) questions. The school environment condition questionnaire was based on Anggriani (2018) and comprised 18 items: 14 positive statements (items 1, 2, 5, 6, 7, 8, 9, 10, 12, 14, 15, 16, and 18) and four negative statements (items 3, 4, 11, 13, and 17), developed from theoretical frameworks. The questionnaire employed the Guttman scale, where positive responses were coded as (1) for “Yes” and (0) for “No,” while negative responses were coded as (1) for “No” and (0) for “Yes.” The questionnaire demonstrated high validity, with a content validity index of 1, assessed through expert evaluation. The selection of items was based on an extensive literature review and adapted from validated instruments used in previous studies on school environmental conditions and Dengue Hemorrhagic Fever (DHF) incidence. A panel of three public health and epidemiology experts reviewed the questionnaire to ensure the relevance and clarity of each item in measuring the intended constructs. Additionally, strong reliability was confirmed with a Cronbach’s alpha coefficient of 0.81, indicating internal consistency and robustness of the instrument.

### Data Collection Procedures

Ethical approval for this study was obtained from the Research Ethics Committee of the Deli Husada Deli Tua Health Institute (Approval No: 091/KEP-IKDH/VII/2023, dated July 3, 2023). The study was conducted between August and October 2023. Participants were briefed on the study’s objectives, benefits, and procedures before data collection. A lottery system was used to randomly select participants. Those who agreed to participate provided written informed consent. Prior to completing the questionnaire, respondents received detailed instructions and were encouraged to seek clarification on any unclear items. Upon completion, the researcher reviewed the questionnaires for completeness, and any missing responses were immediately addressed.

### Data Analysis

Data analysis was performed using SPSS. Bivariate analysis was conducted using the Chi-square (χ²) test to assess the association between school environmental conditions and DHF incidence. Multivariate analysis was performed using logistic regression to identify the most influential variables contributing to DHF incidence. A significance level of p < 0.05 was set for all statistical analyses. Variables with p-values < 0.25 were included in the logistic regression model, which was conducted in two iterations to determine the most significant predictors of DHF incidence.

## Results

### Respondent Characteristics

To provide a clearer understanding of the study sample, Table 1 presents the demographic and environmental characteristics of the respondents. The table includes details such as gender distribution, age groups, grade levels, and school environmental factors, which are essential in analyzing their association with Dengue Hemorrhagic Fever (DHF) incidence.

Based on table 1. Based on gender, the majority of respondents were male 73 people (57.9%). It can be seen that the highest age of student respondents was >10 years old, 85 people (66.70%), and Based on class characteristics, the most respondents were class 4, 51 people (40.40%). Majority the lighting was not eligible 77 (61.40%), humidity was not eligible 71 (56.10%), temperature was not eligible 68 (54.4%), Glass vent was not eligible 77 (61.20%), Dengue fever incidence was 86 (68.4%).

Table 2 presents the statistical relationship between school environmental conditions and DHF incidence, highlighting the impact of inadequate lighting, high humidity, elevated temperatures, and poor ventilation on disease transmission.

**Table 2:** The Relationship Between School Environment Condition and Dengue Hemorrhage Fever Incidence at Public Elementary School (n=126)

Variables	dengue fever	Dengue No	Relative Risk	95%CI	*p* -value
**Lighting**					
Not eligible	64 (82.90)	13 (17.10)	5.80	1.72–19.55	0.00
Qualify	10 (45.50)	13 (54.50)
**Humidity**					
Not eligible	59 (84.40)	11 (15.60)	5.85	1.70–20.12	0.00
Qualify	27 (48.00)	29 (52.00)
**Temperature**					
Not eligible	55 (81.00)	13 (19.00)	3.57	1.00–11.63	0.04
Qualify	31 (53.80)	27 (46.20)
**Glass Vent**					
Not eligible	67 (86.00)	13 (14.00)	10.07	2.76–36.65	0.00
Qualify	17 (38.00)	29 (62.00)			

Based on table 2, it can be seen that the majority of lighting is in the category that does not meet the requirements, as many as 64 people (82.90%). The most dampness was in the category of not meeting the requirements as many as 59 people (84.4%). The highest temperature was in the ineligible category as many as 55 people (81.00%). The most common gauze ventilation category was none, as many as 67 people (86.00%).

Table 3 presents the logistic regression analysis, identifying the most influential environmental factors associated with Dengue Hemorrhagic Fever (DHF) incidence in schools.

**Table 3:** Logistic Regression

Variables	Odds Ratio	95%CI	*p-value*
Humidity	7.16	2.09–38.37	0.00
Glass Vent	18.12	3.36–56.70	0.00

Based on Table 3, humidity and glass ventilation were the most influential variables affecting the incidence of dengue hemorrhagic fever, as determined by logistic regression analysis. Both variables were statistically significant (p < 0.05), indicating a strong association with dengue incidence. Specifically, humidity (OR = 7.16; 95% CI, 2.09–38.37) and glass ventilation (OR = 18.12; 95% CI, 3.36–56.70) demonstrated high odds ratios, suggesting a substantial impact on disease transmission.

The high odds ratio for glass ventilation may be attributed to the fact that inadequate ventilation provides an optimal environment for Aedes mosquito breeding and survival. Poorly ventilated classrooms and buildings may retain humidity and warmth, which are conducive to mosquito proliferation. Furthermore, limited air circulation can increase the likelihood of mosquitoes remaining indoors, thus raising the risk of human-mosquito contact and dengue transmission.

Similarly, high humidity levels create favorable conditions for Aedes mosquitoes, as they rely on moisture for their survival and breeding. Excessive humidity may contribute to prolonged mosquito lifespans and increased viral transmission potential. Additionally, stagnant water accumulation, which is more common in humid environments, serves as an ideal breeding site for mosquito larvae, further exacerbating the risk of dengue fever outbreaks.

## Discussion

In this study, we found a significant relationship between lighting and the incidence of dengue fever (p-value = 0.008). The Odds Ratio (OR) calculation showed a value of 5.8, indicating that lighting levels below the required standard of <120 lux increase the risk of Dengue Hemorrhagic Fever (DHF) infection by 5.8 times compared to adequate lighting. This finding aligns with the study conducted by Liliandriani et al. (2022), which reported a significant association between lighting and DHF incidence (p-value = 0.002). The study suggested that inadequate lighting facilitates mosquito breeding, as mosquitoes tend to thrive in poorly lit environments. Similarly, Fitria (2021) found a significant relationship between lighting and DHF incidence (p-value = 0.008). However, this study contrasts with Mawaddah et al. (2022), who reported no significant relationship (p-value = 1.000). Mawaddah’s study suggested that mosquitoes are more attracted to areas with lighting below 60 lux, which may influence mosquito behavior and habitat preference (Mawaddah et al., 2022; Pakaya et al., 2023; Carneiro et al., 2023).

This study demonstrates a significant relationship between humidity and the incidence of dengue fever, with a p-value of 0.008. The Odds Ratio (OR) calculation yielded a result of 5.85, indicating that inadequate humidity levels increase the risk of Dengue Hemorrhagic Fever (DHF) by 5.85 times compared to adequate humidity. These findings are consistent with the study by Astuti et al. (2020), which reported a significant association between humidity and DHF incidence (p-value = 0.04). Similarly, Widyorini et al. (2017) found a strong relationship between humidity and DHF occurrence. However, this study differs from the findings of Zulfikar et al. (2015), who reported no significant relationship between humidity and DHF incidence.

This research shows that there is a relationship between humidity and the incidence of dengue fever with a p-value of 0.045. The Odds Ratio (OR) calculation resulted in a value of 3.571, indicating that inadequate temperature regulation increases the risk of Dengue Hemorrhagic Fever (DHF) by 3.571 times compared to environments with proper temperature control. This finding aligns with the study conducted by Fitriana and Yudhastuti (2021), which found a significant relationship between temperature and DHF incidence (p-value = 0.019). Similarly, Mulyani et al. (2020) reported a significant association between temperature and DHF incidence (p-value = 0.017). However, these findings contrast with the study by Ghina et al. (2019), which found no significant relationship (p-value = 0.920) between temperature and DHF incidence.

In this study, we also found a significant relationship between gauze ventilation and DHF incidence, with a p-value of 0.001. The Odds Ratio (OR) calculation resulted in a value of 10.075, meaning that the absence of gauze ventilation increases the risk of contracting DHF by 10.075 times compared to the presence of gauze ventilation. This result is consistent with the findings of Liliandriani et al. (2022), who reported a significant association between gauze ventilation and DHF incidence (p-value = 0.004). Similarly, Sucinah et al. (2021) found a significant relationship between gauze ventilation and DHF incidence (p-value = 0.039). However, this study contradicts the findings of Dewi and Sukendra (2019), which found no significant relationship between gauze ventilation and DHF incidence (p-value = 0.135). A poorly ventilated building or school without wire mesh or strimin allows mosquitoes to enter, bite humans, rest, and breed, thereby increasing the risk of DHF transmission (Dewi and Sukendra, 2019; Costas et al., 2023).

## Conclusion

The incidence of dengue fever is influenced by several environmental factors. Effective prevention measures include improving environmental sanitation, eliminating mosquito breeding sites, and ensuring proper waste disposal. In school environments, maintaining adequate lighting, controlling humidity, regulating indoor temperature, and installing protective mesh on ventilation openings are crucial interventions to reduce the risk of dengue transmission. These environmental conditions should be systematically monitored and improved as part of dengue prevention strategies in schools and communities.

## Conflict ofinterests

The authors declare that there is no conflict of interest associated with this study.

**List of Abbreviations :**
DHF : Dengue Hemorrhagic Fever;
OR : Odds Ratio;
PR: Prevalence Ratio;
CI : Confidence Interval
